# RETRACTED: Saadh et al. A Mendelian Randomization Analysis Investigates Causal Associations between Inflammatory Bowel Diseases and Variable Risk Factors. *Nutrients* 2023, *15*, 1202

**DOI:** 10.3390/nu16111778

**Published:** 2024-06-06

**Authors:** Nutrients Editorial Office

**Affiliations:** MDPI, St. Alban-Anlage 66, 4052 Basel, Switzerland; nutrients@mdpi.com

The *Nutrients* Editorial Office retracts the article, “A Mendelian Randomization Analysis Investigates Causal Associations between Inflammatory Bowel Diseases and Variable Risk Factors” [1], cited above.

Following publication, concerns were brought to the attention of the publisher regarding the authenticity of the authorship of this article [1].

Adhering to our complaint procedure, an investigation was conducted by the Editorial Office and Editorial Board. Following communications with the authorship group and related institutions, the Editorial Office was unable to verify the identity, contribution, or affiliations of a number of the authors listed on this manuscript, nor could the origins of the study be confirmed. As a consequence, the Editorial Board has decided that they no longer have confidence in the integrity of the findings and have decided to retract this publication [1] as per MDPI’s retraction policy (https://www.mdpi.com/ethics#_bookmark30 (accessed on 6 May 2024)).

This retraction was approved by the Editor-in-Chief of the journal *Nutrients*.

The authors did not provide a comment on this decision.
